# Histone demethylases UTX and JMJD3 are required for NKT cell development in mice

**DOI:** 10.1186/s13578-017-0152-8

**Published:** 2017-05-17

**Authors:** Daniel Northrup, Ryoji Yagi, Kairong Cui, William R. Proctor, Chaochen Wang, Katarzyna Placek, Lance R. Pohl, Rongfu Wang, Kai Ge, Jinfang Zhu, Keji Zhao

**Affiliations:** 1 Systems Biology Center, National Heart, Lung and Blood Institute, National Institutes of Health (NIH), Bethesda, MD 20892 USA; 2 Laboratory of Immunology, National Institute of Allergy and Infectious Diseases, NIH, Bethesda, MD 20892 USA; 3 Center of Immunology, National Heart, Lung and Blood Institute, NIH, Bethesda, MD 20892 USA; 4 Laboratory of Endocrinology and Receptor Biology, National Institute of Diabetes and Digestive and Kidney Diseases, NIH, Bethesda, MD 20892 USA; 5 Departments of Pathology and Immunology, Center for Cell and Gene Therapy, Dan L. Duncan Cancer Center, Baylor College of Medicine, Houston, TX 77030 USA

## Abstract

**Background:**

Natural killer (NK)T cells and conventional T cells share phenotypic characteristic however they differ in transcription factor requirements and functional properties. The role of histone modifying enzymes in conventional T cell development has been extensively studied, little is known about the function of enzymes regulating histone methylation in NKT cells.

**Results:**

We show that conditional deletion of histone demethylases UTX and JMJD3 by CD4-Cre leads to near complete loss of liver NKT cells, while conventional T cells are less affected. Loss of NKT cells is cell intrinsic and not due to an insufficient selection environment. The absence of NKT cells in UTX/JMJD3-deficient mice protects mice from concanavalin A‐induced liver injury, a model of NKT‐mediated hepatitis. GO‐analysis of RNA-seq data indicates that cell cycle genes are downregulated in UTX/JMJD3-deleted NKT progenitors, and suggest that failed expansion may account for some of the cellular deficiency. The phenotype appears to be demethylase‐dependent, because UTY, a homolog of UTX that lacks catalytic function, is not sufficient to restore their development and removal of H3K27me3 by deletion of EZH2 partially rescues the defect.

**Conclusions:**

NKT cell development and gene expression is sensitive to proper regulation of H3K27 methylation. The H3K27me3 demethylase enzymes, in particular UTX, promote NKT cell development, and are required for effective NKT function.

**Electronic supplementary material:**

The online version of this article (doi:10.1186/s13578-017-0152-8) contains supplementary material, which is available to authorized users.

## Background

T cell development occurs in the thymus and proceeds through several immature stages. Committed T progenitors rearrange a T cell receptor (TCR) and express CD4 and CD8 co-receptors at the double positive (DP) stage. Specific patterns of TCR signaling direct development toward one lineage [1]. Most mature cells are either CD4^+^ helper T cells or CD8^+^ cytotoxic T cells, though DP cells also generate natural killer T (NKT) cells, a distinct population that shares the properties of T cells and natural killer (NK) cells [2].

NKT cells recognize lipid rather than peptide antigens, and are enriched in the liver. Many NKT cells utilize a characteristic Vα‐Jα rearrangement with limited TCRβ repertoire. This TCR can be stimulated by a lipid molecule, α‐Galactosyl ceramide (αGalCer), presented by CD1d, and is selected on self‐lipid‐CD1d determinants [3]. NKT cells also have distinct functional properties. They are capable of rapid secretion of a wide variety of cytokines [4]. Because of their fast action and access to the blood stream they are important cellular components of pathogenic inflammation in the liver and lung, and also fight cancer and infection during innate immune responses [3, 5].

These distinct properties result from the NKT specific transcriptional program [6]. T cell transcription factors T‐bet [7, 8], ID2 [9], and RORγt [10] are essential for NKT cell development. While these factors are shared with conventional T cells, the transcription factor PLZF is a more restricted [11]. The gene that codes PLZF, Zbtb16, is a direct target of Egr1 and Egr2, which are induced at high levels after strong signaling through the TCR [12]. Thus, Egr2 is also required for iNKT development [13]. When c‐myc is deleted using CD4‐Cre, conventional T cells development is normal, but NKT cell development arrests in the thymus [14].

UTX [15–18] and JMJD3 [19] are closely related histone demethylases that act specifically on di- and tri‐ methylated lysine 27 of histone H3 (H3K27me2,3). Since these methylations are associated with gene repression, removal of these marks by UTX and/or JMJD3 may result in gene activation. UTX interacts with protein complexes that are associated with H3K4 methylation (a mark of active transcription) by MLL family proteins [17, 20] and nucleosome remodeling activity by the recruitment of BRG1 [21]. JMJD3 associates with members of the transcriptional elongation complex [22]. Because the proteins associated with UTX and JMJD3 possess multiple enzymatic activities involved in chromatin modification, the UTX/JMJD3 containing complexes may be potent transcriptional activators.

Deletion of *Kdm6a*, the gene that encodes UTX, causes embryonic lethality [23]. Conditional loss-of‐function studies demonstrated that UTX is necessary in heart development, and participates in WNT signaling during mesodermal differentiation [23, 24]. Developing muscle utilizes UTX to remove H3K27me3 from the gene body regions of newly expressed genes [25, 26]. Similarly, deletion of *Kdm6b*, the gene that encodes JMJD3, causes embryonic lethality [27]. Jmjd3 is an important regulator of inflammation in macrophages [19], and is critical for type 2 macrophage polarization [27, 28]. In mature T cells, UTX and JMJD3 can also interact with T‐bet and Eomes to activate gene transcription [21, 29].

Work from many groups has demonstrated that the histone modification profiles are tightly correlated with gene activity [30–36]. In order to perturb H3K27 methylation we deleted UTX and JMJD3 in developing T cells using Cre recombinase driven by CD4 promoter elements (CD4-Cre). Surprisingly, we found a dramatic reduction of NKT cells but only modest defects in conventional T cells in UTX and/or JMJD3-deleted mice.

## Methods

### Mice and cell preparation

UTX and Ezh2 floxed mice were provided by Kai Ge. Ezh2‐floxed mice were generated by Alexander Tarakhovsky. Kdm6b‐floxed mice were a gift from Rongfu Wang. CD4‐cre and C57BL/6 mice were purchased from Taconic and Jackson, respectively. C/57BL6.CD45.1 congenic mice were purchased from the NCI-Frederick mouse repository.

To isolate liver lymphocytes, the liver was perfused with PBS and then passed through a mesh filter to create a single cell suspension. The single-cell suspension was resuspended in 35% Percoll in RPMI with 100 U/ml of heparin and centrifuged 15 min at 2000 RPM. The pelleted cells were analyzed by flow cytometry. Splenocytes, lymph node cells, or thymocytes, were isolated by standard procedures. RBCs were lysed with ACK lysing buffer when needed. All procedures were approved by NHLBI animal committees.

To create bone marrow chimeric mice bone marrow from donor mice was depleted of CD19^+^ and GR-1^+^ by antibody labeling and magnetic selection using goat α-Rat IgG beads (Qiagen). WT CD45.1 depleted bone marrow cells were mixed with depleted bone marrow cells from Cre positive (UTX/JMJD3 DKO) or Cre negative (WT control) CD45.2 UTX^fl/fl^JMJD3^fl/fl^ mice at a 1:1 ratio. WT CD45.1 host mice were prepared by whole body lethal irradiation (900 rad, cesium source). Six hours after irradiation 2 million mixed bone marrow cells were injected I.V. via the retro-orbital sinus. Sulfatrim antibiotic was supplied in the drinking water until mice were sacrificed 5–7 weeks later.

For BRDU incorporation, mice were given a 100 μl intraperitoneal injection of 10 mg/ml BRDU solution in PBS. Twelve hours later mice were sacrificed and organs harvested. Following surface staining, thymocytes were permeablized and treated with DNAse. Finally they were stained with α-BRDU antibodies and DAPI and analyzed by FACS.

### FACS and western blot

Flow cytometry and western blot were performed by standard procedures. Tbet staining was done using the eBioscience FoxP3 fix and permeabilization buffers and the manufacturers protocol. All plots are gated for light scatter on lymphocytes, and except for experiments were permeabilization was done dead cells are excluded by gating on cells that do not incorporate DAPI or 7-AAD. The antibodies and reagents with the supplier are listed in Additional file 1: Table S1. Data were collected on a FACSCanto or LSRII. For sorting experiments, a FACSAria2 was used. Data were analyzed using FlowJo. Frequency plots and t tests were done with Prism software. The two-tailed T test was used to test statistical significance.

### RNA-seq and data analysis

To isolate RNA from NKT precursors cells were sorted directly into Trizol lysis buffer, and RNA was isolated according to manufacturer protocols. Double stranded DNA for solexa sequencing was created from one hundred to five hundred cell equivalents of RNA by adhering closely to a published protocol [37]. Briefly, total RNA was resuspended in water at 1000 cell equivalents per μl based on counts from the cell sorter. According to the protocol cited, this RNA was denatured, and reverse transcription was performed by using a tagged poly-T (P1) primer. The RNA was then digested, and TDT was used to add a second A-tail to the 3′ end of the cDNA. Second strand synthesis was performed by using a second tagged poly-T (P2) primer and then free primer was digested with exonuclease 1. The resulting double stranded cDNAs were amplified by primers complimentary to unique regions of P1 and P2 by PCR. A second PCR is done using 5′ amine blocked primers which inhibit ligation to these ends in subsequent steps. Libraries with successful amplification that were considered for further analysis had a size range of the resulting cDNA from 500 to 3000 bp with fairly equal representation. If there is too much starting material smaller 500 to 1000 bp fragments dominate.

The resulting double stranded DNA was sonicated to 150–300 bp by sonicating in a Diagenode BioRuptor. Following sonication, 1–2 μg the DNA was prepared for Solexa HiSeq. Briefly, blunt ends were generated by End-It (Epicenter) end repair kit according to the manufacturer’s protocol. Single A’s were added to the DNA by incubation with truncated klenow fragment (NEB) and Solexa adaptors were ligated to the A-tailed fragment by DNA ligase (NEB). Finally, 6 bp indices were introduced by PCR with the indexed primer kit from Illumina. All samples were sequenced at the NHLBI sequencing facility.

In order to analyze the gene expression from this RNA-seq, sequence tags were aligned to the mm9 genome using Bowtie aligner [38], allowing 2 mismatches per tag. Reads per kilobase of message (RPKM) per million reads was calculated for each gene using software written by Iouri Chepelev [39]. Using exon locations from ensembl database, the software counts the number of tags in the exons of each gene. RPKM is then the number of exon reads per gene, normalized to the size of the gene and to the size of the library. Further analysis of gene expression was done using Excel. Both samples WT and UTX/JMJD3 DKO NKT precursor cells were analyzed in triplicate. Biological replicates were prepared from single mice in pairs. We analyzed four female mice (2 WT, 2 DKO) and two DKO mice (1 WT, 1 DKO). Paired samples were analyzed for genes with 2.5-fold differences in gene expression. We focused on genes that had this difference (2.5 fold) in all 3 samples and had RPKM of at least 3 in all three samples with higher expression (i.e. RPKM >3 in all WT samples for a gene that is higher in WT cells). Promoter motif analysis for the differentially regulated genes was done using the FindMotifsGenome program in the Homer program package. Raw data are available as GSE47081 in the Geo database.

### Concanavalin A model of liver injury

Concanavalin A-mediated liver injury was performed using established methods with minor deviations [40]. Briefly mice were injected intravenously via tail‐vein with 12.5 mg/kg of concanavalin A (Type V, Sigma‐Aldrich, St. Louis, MO) dissolved in sterile PBS at a final concentration of 1.25 mg/ml. Serum samples were taken at 8 h post injection. Liver injury was evaluated by histopathological examination of hematoxylin‐ and eosin‐ (H&E) stained, formalin‐fixed, paraffin‐embedded liver sections (American Histolabs, Gaithersburg, MD) by light microscopy examination and by serum activity of alanine aminotransferase (ALT) measured with a diagnostic kit (Teco Diagnostics, Anaheim, CA).

## Results

### Deletion of UTX and JMJD3 caused a global increase in H3K27me3

Mice with conditional mutant alleles of *Kdm6a* encoding UTX or *Kdm6b* encoding JMJD3 were bred to mice expressing CD4-cre to generate *Kdm6a* ^fl/fl^-CD4Cre (CD4-specific UTX KO, referred to as UTX KO) or *Kdm6b* ^fl/fl^-CD4Cre (CD4-specific JMJD3 KO, referred to as JMJD3 KO) mice. To investigate redundant functions of UTX and JMJD3, double knockout (*Kdm6a* ^fl/fl^ *Kdm6b* ^fl/fl^-CD4Cre, CD4-specific UTX/JMJD3 DKO, referred to as UTX/JMJD3 DKO) mice were also created.

To determine whether loss of UTX and JMJD3 would affect the global levels of H3K27 di- or tri-methylation (H3K27me2, H3K27me3) in T cells we determined H3K27 methylation levels in CD4^+^ splenocytes by western blot. We found that H3K27me3 was elevated after loss of both enzymes but not in single mutant mice (Additional file 2: Figure S1). Quantification of the Western blotting from three biological replicate samples indicated that the H3K27me3 signal increased approximately twofold after deletion of both UTX and JMJD3 (Additional file 2: Figure S1).

### T cell development is slightly altered after the loss of UTX, JMJD3 or both

**Fig. 1:**
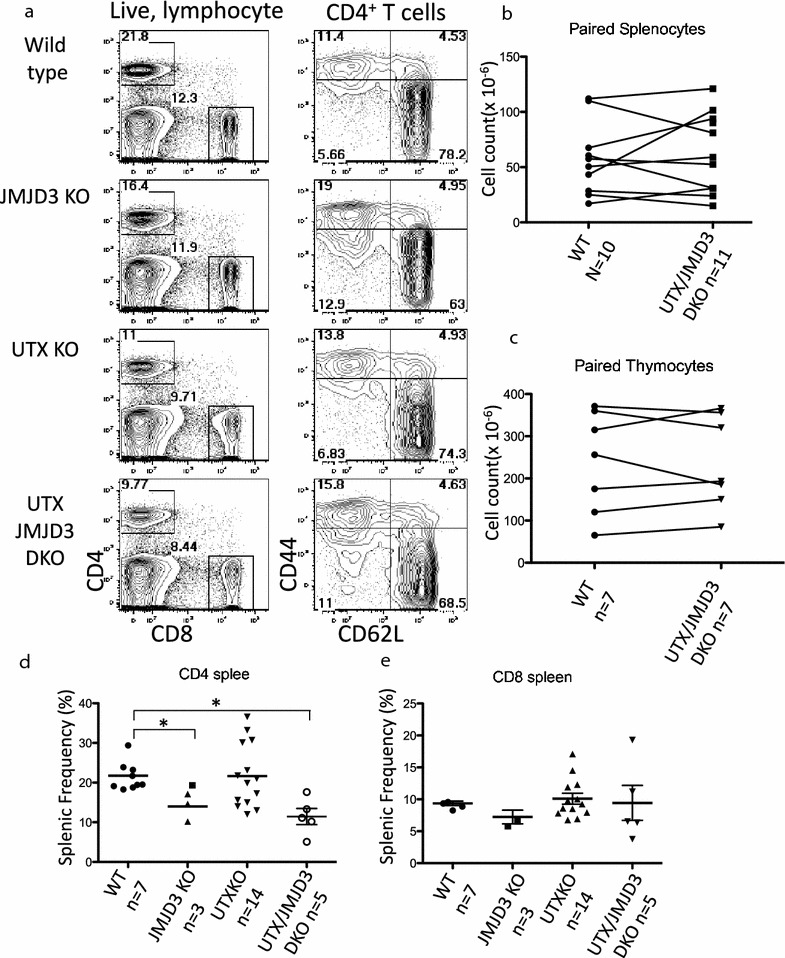
Loss of UTX and JMJD3 partially inhibits CD4 development, but CD8 T cells are intact. **a** Representative staining of splenocytes from WT, JMJD3, UTX and UTX/JMJD3 DKO mice were isolated and stained with the indicated* markers* of conventional T cells. Numbers of animals are indicated in **d**, **e**, and were analyzed in 3 separate experiments. **b**, **c** Cell counts from the spleens and thymi of WT and age matched litter mate control mice. Although cell counts vary across age, there is no reproducible difference between the genotypes in a given experiment. **d**, **e** CD4 and CD8 frequencies in the spleens of mice from the indicated genotypes. There is no significant difference between WT and UTX KO CD4 cells while JMJD3 and UTX/JMJD3 show statistically reduced CD4 frequency (two-tailed T test, p < 0.05). CD8 T cell frequency is unaffected by loss of these genes

To examine whether UTX and JMJD3 contribute to T cell development, we analyzed the CD4 and CD8 T cell populations from the spleens of the three knockout mouse strains by flow cytometry (Fig. 1a, left panels). We note that loss of UTX led to reduced levels of CD44, particularly on CD62L^+^ cells (Fig. 1a, right panels). Further separating CD4^+^ T cells by CD44 and CD62L revealed that memory (CD44^+^CD62L^−^) and naïve (CD44^−^CD62L^+^) cells were present at normal levels (Fig. 1a, right panels). The mice we analyzed in these experiments varied in age from 5 weeks to 4 months, and each experiment was performed with age matched litter-mate control animals. While we found that the cell counts varied between experiments, there was no reproducible difference between WT and UTX/JMJD3 DKO in the number of cells in the spleen or thymus (Fig. 1b, c).

Because the cell counts were not directly comparable between experiments we analyzed the frequency of CD4 (Fig. 1d) and CD8 (Fig. 1e) T cells and found that there was statistically significant difference in the frequency (p < 0.05) of CD4 cells in DKO mice (Fig. 1d). There was also a significant reduction of CD4 T cells in JMJD3 mice (p < 0.05), however due to more pronounced differences described below we chose to first analyze the DKO mice. The frequency of CD8 T cells was not significantly affected by the loss of these enzymes. Thus, the loss of UTX and JMJD3 only modestly affects conventional T cell development.

### NKT cell frequency is greatly reduced in the livers of UTX and UTX/JMJD3 DKO mice

**Fig. 2:**
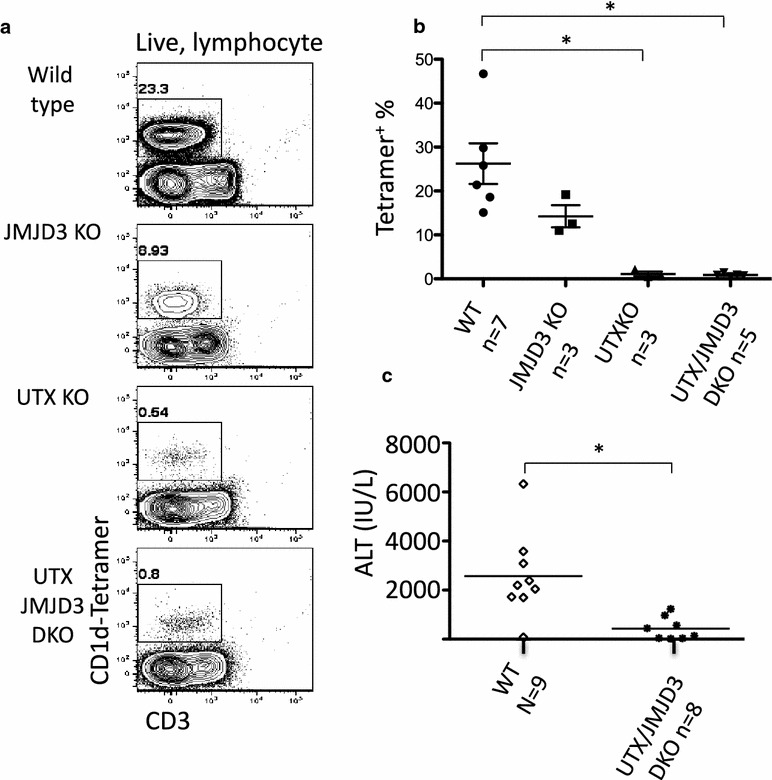
UTX and UTX/JMJD3 deficient mice lack NKT cells in the liver. **a** Liver lymphocytes were isolated from female WT, JMJD3 KO, UTX KO, and UTX/JMJD3 DKO mice and analyzed for CD1d tetramer and CD3e by FACS. **b** Quantification of the data presented in **a** representing data collected in 3 separate experiments. By two-tailed T test, the difference between WT and UTX KO (p < 0.01) and WT and UTX/JMJD3 DKO (p < 0.005) mice is significant. The difference between WT and JMJD3 is not significant (p = 0.11). **c** Mice were injected with ConA and serum was collected 8 h later. Serum was measured for the presence of ALT which is an indication of liver damage. These results were collected in two experiments with 9 (WT) and 8 (DKO) mice. The difference between WT and UTX/JMJD3 DKO mice is significant (two-tailed T test, p < 0.05)

To investigate NKT cells we isolated liver lymphocytes from female wild type (WT) or CD4-specific UTX KO, JMJD3 KO, or UTX/JMJD3 DKO mice. As expected, a large percentage of liver lymphocytes were tetramer^+^ iNKT cells in WT mice (Fig. 2a). Loss of JMJD3 resulted in a 50% reduction in the percentage of tetramer-binding iNKT cells among liver lymphocytes (Fig. 2a). The loss of UTX in female mice (with or without JMJD3) led to a near complete loss of NKT cells (Fig. 2a). The results from multiple samples are summarized in Fig. 2b. Thus, JMJD3 and UTX contribute to the generation of liver NKT cells, and UTX has a greater role than JMJD3. Because the phenotype caused by loss of JMJD3 was milder we focused on the role of UTX.

### Loss of iNKT cells in UTX/JMJD3 DKO mice protects against concanavalin A-induced liver damage

To determine whether there was a functional consequence by the absence of NKT cells in UTX/JMJD3 DKO mice and that the absence of cells was not simply a change in surface marker expression, we employed the concanavalin A (ConA)-mediated liver hepatitis model. Following IV injection of ConA, NKT cells are among the first cells to respond to this stimulus, and their cytokine release causes activation and recruitment of neutrophils and eosinophils because of their position near the liver vasculature. NKT cells are required for ConA-induced liver damage since CD1d deficient mice, which lack iNKT cells [41], are protected against ConA-induced liver damage [40].

Following ConA injection, the spleen was enlarged in all animals (WT or DKO), demonstrating that UTX and JMJD3 are not necessary for a response to ConA (not shown). We measured serum alanine aminotransferase, which is an indicator of liver damage, and found that there is significantly less ALT in the serum of DKO animals than in WT littermate controls (Fig. 2c). Thus, in addition to the loss of phenotypic NKT cells, mice with deletion of UTX/JMJD3 also have reduced NKT function, although we cannot completely rule out the possibility that other cells may also be involved in the process.

### Thymic NKT development in UTX/JMJD3 DKO cells is arrested at an immature stage

The lack of NKT cells in the liver could result from developmental arrest in the thymus or a homing defect. To determine if a developmental block led to the decrease of mature NKT cells in the liver, we isolated thymocytes from WT or CD4-specific UTX KO, JMJD3 KO, or UTX/JMJD3 DKO mice.

Developing NKT cells can be identified in the thymus because they bind to a CD1d-α-GalCer tetramer reagent. Tetramer-positive NKT cells transit four developmental stages marked by the expression of CD24, CD44 and NK1.1. P0 cells express CD24, and all subsequent stages do not express this surface marker. P1 cells do not express either marker (CD1d-tetramer^+^CD44^−^NK1.1^−^), P2 cells express CD44 and not NK1.1 (CD1d-tetramer^+^CD44^+^NK1.1^−^), and P3 cells are mature iNKT cells that express both markers (CD1d-tetramer^+^CD44^+^NK1.1^+^) [5].

**Fig. 3:**
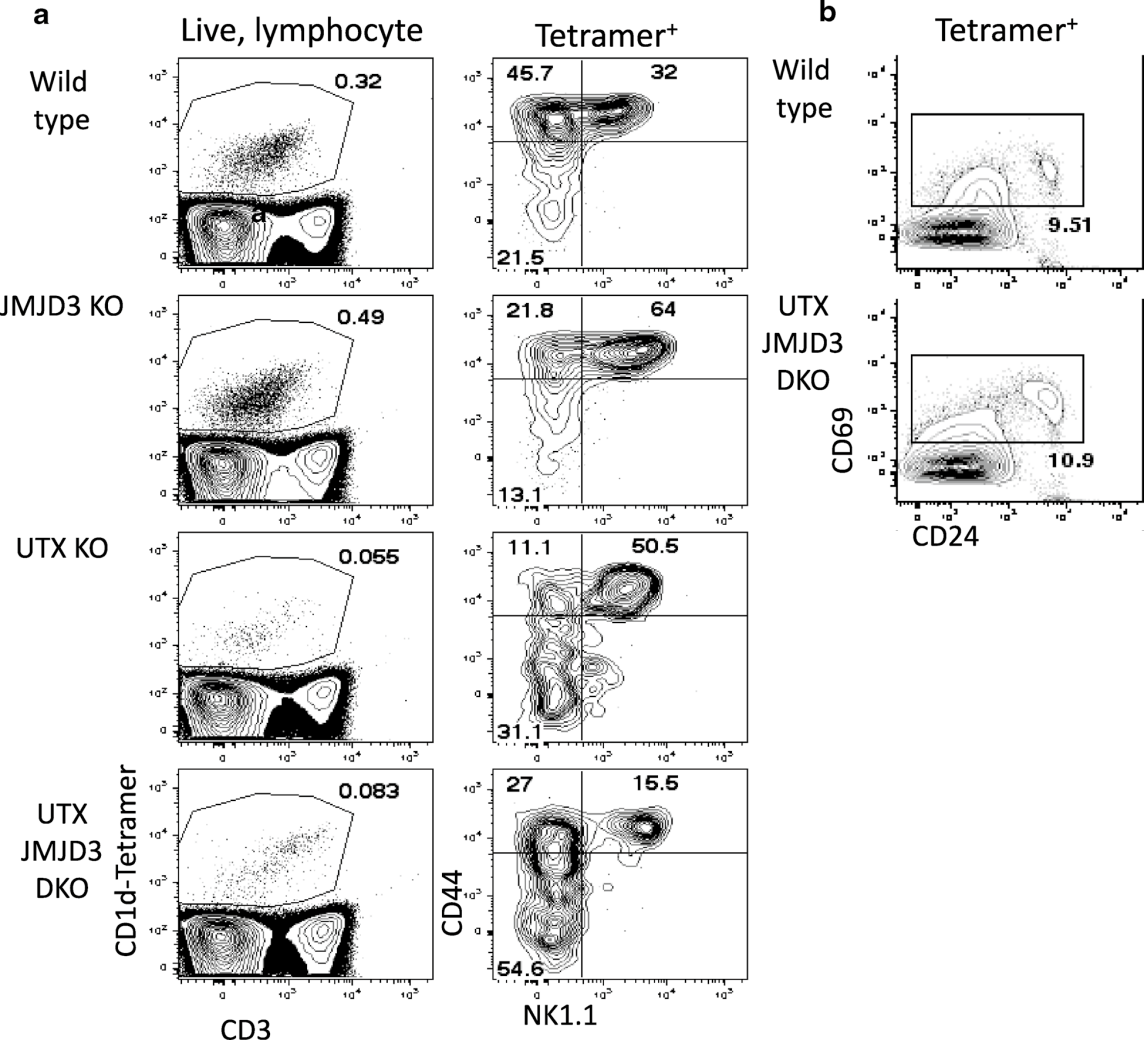
UTX promotes thymic NKT maturation at the P1–P2 stage. **a** Thymocytes were isolated from female WT, JMJD3 KO, UTX KO, and UTX/JMJD3 KO mice. Cells were stained with CD1d tetramer reagent and other markers of NKT precursors. *Plots* were gated by light scatter on lymphocytes (*left panels*) or tetramer^+^ cells (*right panels*). Results are representative of 3 or more mice per group in 3 experiments. **b** Tetramer^+^ thymocytes were selected by magnetic enrichment and stained for CD69 and CD24. This* plot* shows that NKT P0 is intact and that these cells express CD69 in DKO mice. This stain was done on 3 WT and 3 DKO mice

Investigation of tetramer-positive cells in the thymus of WT and JMJD3 mutant mice revealed little difference between tetramer-binding cells after deletion of JMJD3 (Fig. 3a). We observed a 50–90% reduction in tetramer binding cells in the thymi of UTX KO and UTX/JMJD3 DKO mice (Fig. 3a). In UTX/JMJD3 DKO mice we found that many fewer tetramer-binding cells expressed CD44 or NK1.1, with more CD44^−^NK1.1^−^ P1 cells (Fig. 3a, right panels). Our finding that CD44 expression is down-regulated in UTX-deficient CD4 T cells confounds conclusions about the exact developmental block because this marker normally defines the P1 to P2 transition. Regardless, the developmental block is not simply a result of a failure to express CD44, because deletion of UTX does not lead to the appearance of NK1.1^+^CD44^−^ cells (otherwise mature NKT, lacking CD44). The deficiency of NKT cells in UTX/JMJD3 likely results from this developmental block.

Next, we investigated whether there were additional defects before the P1 to P2 transition. NKT P0 cells are extremely rare and so we enriched tetramer positive cells by MACS enrichment. By further characterizing these cells from DKO mice we found that UTX/JMJD3 DKO mice have CD24 P0 cells. Furthermore, most of these cells express CD69, which indicates that the DKO cells have received signals through the TCR (Fig. 3b). These results indicate that the defect in NKT cell development is not caused by a failure of TCR signaling.

### The requirement for UTX and JMJD3 in NKT cells is cell intrinsic

**Fig. 4:**
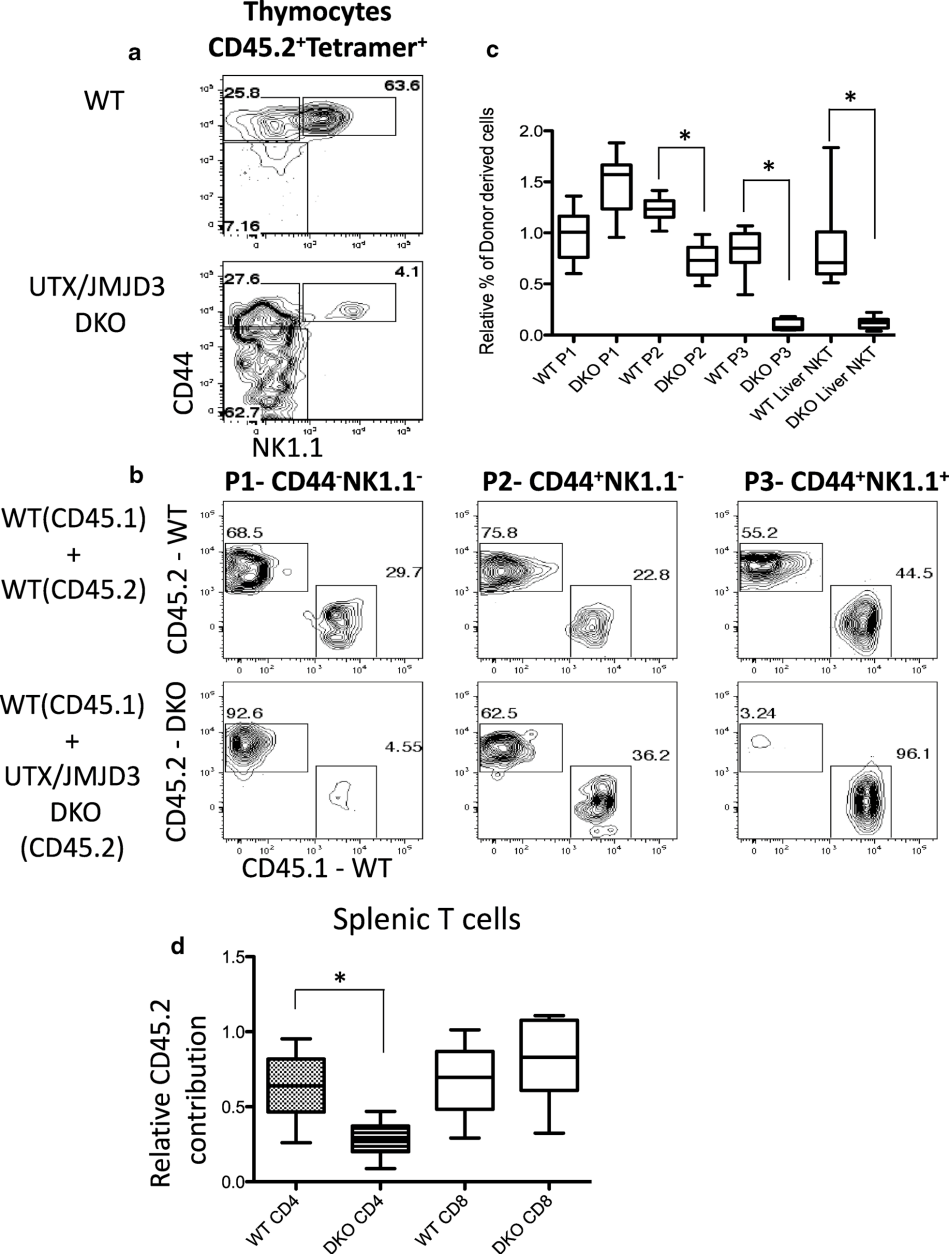
The requirement for UTX and JMJD3 in NKT cell development is cell intrinsic. Thymocytes were isolated from chimeric mice that were reconstituted with bone marrow from WT or UTX/JMJD3 DKO mice mixed with WT CD45.1 congenic bone marrow 5–7 weeks prior to analysis. **a** Thymocytes were stained with CD1d tetramer reagent and other markers of NKT precursors. *Plots* were gated by light scatter on lymphocytes, and dead cells were excluded by DAPI staining. Shown are CD45.2^+^tetramer^+^ cells. **b** Representative plots of donor and host markers are shown for each thymic NKT population P1–P3. **c** Quantification of results from 3 experiments with 10 mice in the WT control and 9 mice in the DKO experimental group. In order to control for reconstitution efficiency, the fraction of the donor population was normalized to the fraction of donor cells in splenic B cells. This serves as a control, because these cells do not express CD4-cre and do not excise the floxed alleles. *Asterisk* indicates p < 0.005 by two-tailed T test. **d** As in **c**, the CD45.2 contribution to splenic CD4 or CD8 T cells was assessed by FACS. The difference between WT and DKO is significant (p < 0.005). Any difference in CD8 reconstitution is not significant

Conventional T cells are selected by non-hematopoietic stromal cells, but NKT cells are selected by CD1d ligands expressed on double positive (DP) thymocytes. Because CD4-cre driven deletion occurs in DP thymocytes it is possible that UTX and JMJD3 regulate essential components of the selection environment. To determine if the presence of WT selecting cells would rescue the deficiency of NKT cells caused by loss of UTX and JMJD3 we created mixed bone marrow chimeras using B6.CD45.2-UTX/JMJD3 DKO bone marrow and B6.CD45.1 congenic bone marrow. We investigated DKO CD45.2^+^ cells in the thymus and found that the developmental block seen in DKO mice also occurs in the presence of a WT selecting environment (Fig. 4a). Control WT B6.CD45.2 bone marrow contributed to all stages of NKT cell development. In contrast, DKO bone marrow made diminishing contributions to the NKT compartment during maturation. By the mature stage, P3, there were almost no cells from the UTX/JMJD3 DKO bone marrow (Fig. 4b). In order to account for differences in reconstitution efficiency and to quantify these results across several animals we normalized the fraction of CD45.2^+^ cells in each NKT precursor population to the fraction of CD45.2 cells among splenic B cells (CD45^+^CD19^+^) in the same animal (Fig. 4c). We note that there is an accumulation of DKO cells at the P1 stage, supporting the conclusion that the developmental arrest caused by loss of UTX and JMJD3 is cell intrinsic.

In order to confirm our previous observations about CD4 and CD8 T cells we checked the contribution of CD45.2^+^ cells to these populations in the spleen. As we observed with unmanipulated mice DKO progenitors were less competent to generate CD4 T cells, while there was no defect in CD8 T cells (Fig. 4d). This defect is not as substantial as that seen for NKT development, but may be an interesting direction for future research.

### UTY does not substitute UTX to support NKT development

The UTX gene is located on the X chromosome and has a homolog on the Y chromosome, UTY. Although the UTX and UTY proteins are structurally similar, UTY has amino acid substitutions that diminish its demethylase activity [18, 42]. Importantly, UTX is bi-allelically expressed in females [43], and both UTX and UTY are expressed in male mice. In other experimental systems, mutant forms of UTX or JMJD3 that lack catalytic activity, or UTY, can substitute for WT UTX for gene activation [21, 24]. To assess whether the demethylase function of UTX is important in the development of NKT cells, we investigated the phenotype of male mice.

**Fig. 5:**
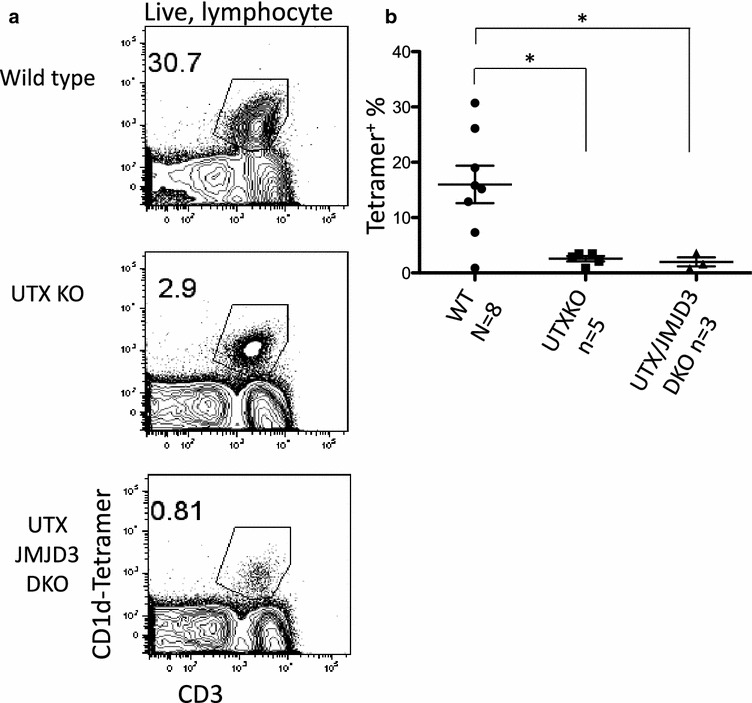
UTY does not support efficient NKT development. **a** Liver lymphocytes were isolated from male WT, UTX KO, and UTX/JMJD3 DKO mice, and analyzed for CD1d tetramer and CD3e by FACS. **b** Quantification of the data presented in **a** representing data collected from 3 separate experiments. By two-tailed T test, the difference between WT and UTX KO (p < 0.05) and WT and UTX/JMJD3 DKO (p < 0.05) mice is significant

We isolated liver lymphocytes from WT or CD4-specific UTX KO, JMJD3 KO and UTX/JMJD3 KO mutant male mice and analyzed NKT cells using flow cytometry. Similar to JMJD3 KO females, JMJD3 KO males (a somatic gene) have a 60% reduction in liver iNKT cells (data not shown). Similar to UTX KO females, the frequency of NKT cells in UTX KO males (UTX^−^UTY^+^) is reduced 90% (Fig. 5a). In order to ensure that the defect was not caused by UTX haplo-insufficiency we compared UTX heterozygous females to knock out males. We found that UTX heterozygous females did not have an NKT defect (data not shown). UTX/JMJD3 DKO males show a similar reduction in the frequency of liver NKT cells (Fig. 5a). In the thymus, UTX/JMJD3 DKO males have the same developmental block seen in female mice. These results indicate that UTX is necessary for NKT development, suggesting a role for the demethylation enzymatic function.

### Deletion of EZH2 partially rescues the NKT developmental defects caused by loss of UTX

**Fig. 6:**
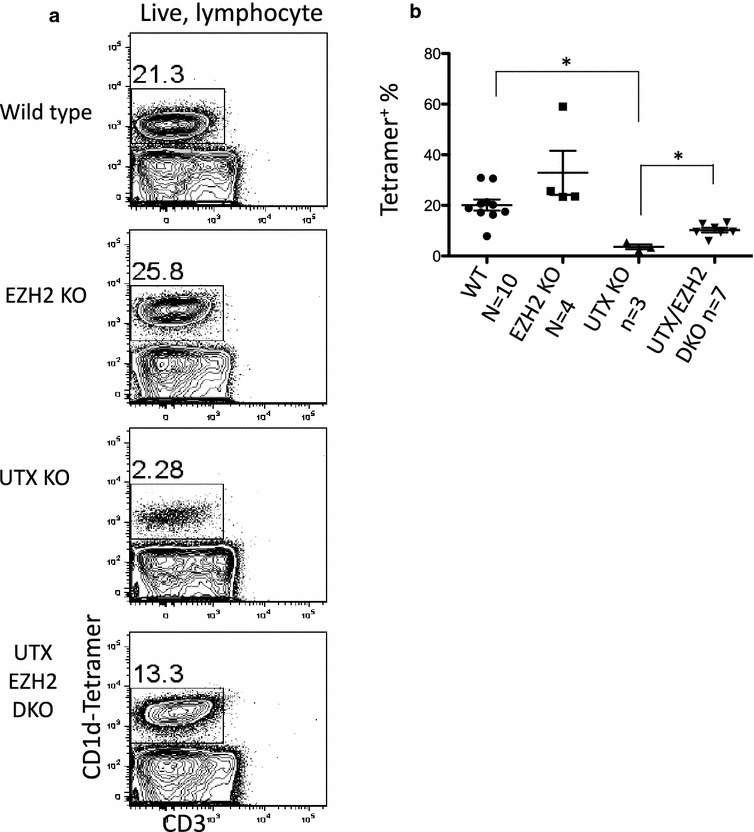
Loss of EZH2 enhances NKT development, and partially rescues the blockage caused by loss of UTX. **a** Liver lymphocytes were isolated from WT, EZH2 KO, UTX KO, and UTX/EZH2 DKO mice, and analyzed for CD1d tetramer binding and CD3e levels by FACS. **b** Quantification of 3 experiments of indicated genotype showing the difference in frequency of tetramer positive cells in the liver. The difference between WT and CD4-specific UTX KO is significant by two-tailed T test (p < 0.02). There is also a significant difference between UTX KO and UTX/EZH2 DKO (p < 0.02), indicating the partial rescue

To gain additional evidence that the loss of NKT cells caused by UTX deficiency is related to H3K27 methylation, we undertook a second genetic approach. EZH2 is the enzymatic component of the Polycomb Repressor Complex 2 (PRC2), which is responsible for the majority of H3K27me3 deposition [44, 45]. To test the genetic interaction between UTX and EZH2 in the generation of liver NKT cells, we generated CD4-specific UTX, EZH2, and UTX/EZH2 double knockout mice. In order to confirm the deletion of EZH2 affected H3K27me3 levels we performed western blot of H3K27me3. The absence of EZH2 in T cells lead to a near complete H3K27me3 as determined by western blot on CD8^+^ T cells (Fig. 6a, b).

We analyzed liver lymphocytes of these mice and found that EZH2-deficient mice have a modest increase in NKT cells in the liver (Fig. 6c). Interestingly, we also found that there was a higher frequency of developing NKT cells in the thymus (Additional file 3: Figure S2), indicating that the reduced deposition of H3K27me3 may lead to an increase in NKT developmental choice. To investigate whether an antagonistic relationship exists between EZH2 and UTX during NKT development, liver lymphocytes from CD4-specific UTX/EZH2 double knockout mice were analyzed. In these mice, NKT cell frequencies were partially restored (Fig. 6c). This result suggests that key regulators of NKT development are silenced by H3K27me3 and silencing activity of EZH2 is antagonized by demethylation of H3K27me3 by UTX.

### Gene expression profiling of NKT progenitors reveals that cell cycle genes and known regulators of NKT development are down regulated in the absence of UTX and JMJD3

To systematically address the mechanism for the developmental block imposed by loss of UTX and JMJD3, we isolated P1 NKT progenitors from the thymi of WT mice and CD4-specific UTX/JMJD3 DKO in triplicate (2 females, 1 male) and profiled their gene expression using RNA-Seq. We focused on the P1 population, because the developmental block appeared to occur in CD4-specific UTX/JMJD3 DKO mice at the P1 to P2 transition. Because the number of NKT progenitor cells is extremely limiting (5000–12,000 cells per WT mouse), we employed a sensitive RNA amplification technique [37]. Short sequence reads were aligned to the mm9 reference genome using Bowtie and quantified as reads per kilobase message (RPKM) for ensemble reference genes. We restricted our analysis to genes that were differentially regulated (twofold), and expressed (RPKM >3) in all 3 samples (WT or DKO) with higher expression.

Because UTX and JMJD3 remove H3K27me3, thus relieving gene repression, we expected that more genes would be higher in WT cells than DKO cells. Accordingly, 121 genes were higher WT samples than the paired DKO counterpart, and 41 were higher in the DKO samples (Additional file 4: Table S2). This list does not include PLZF, Egr1 and Egr2, suggesting UTX and JMJD3 are not involved in regulating the expression of these key transcription factors.

We preformed further analysis on genes that are higher in WT cells, since genes that are activated by the demethylases are more likely the direct targets. We used the HOMER software package [46] for GO analysis and found that the predominant biological function of genes was related to cell cycle.

Based on these data, we hypothesized that WT P1 cells would be in rapid cell cycle, however by staining with the DNA dye DAPI we found that only a small fraction of tetramer positive cells was in cell cycle and that this was not different between WT and DKO mice. We also investigated BRDU incorporation between WT and UTX KO mice. While a minor fraction of the tetramer positive cells did incorporate BRDU, there was no difference between strains. The NKT deficiencies in UTX/JMJD3 DKO mice may be related to cell cycle and a failure to expand, but we have not been able to establish a direct connection (Additional file 5: Figure S3). Future work will determine whether UTX and JMJD3 directly regulate cell cycle in NKT cells or whether they regulate a signaling molecule that allows for expansion of NKT cells.

## Discussion

We found that UTX and JMJD3 are required for the development of NKT cells, while conventional T cell development is less affected. Our genetic evidence indicates that the block in NKT cell development in UTX KO mice is caused by the increase of H3K27 methylation. This is distinct from other biological processes that have been well characterized during mesodermal differentiation as well as some gene expression characterized in T cell lines. In these examples, mutants of UTX that lack catalytic function or UTY can substitute for UTX and are thought to work by recruiting BRG1 and other complex members such as MLL3/4.

For the development of liver NKT cells, UTX plays a more significant role than JMJD3. The reason for the differential requirement of UTX and JMJD3 in NKT development is not clear. Both enzymes are expressed at high levels, and are not differentially regulated during NKT differentiation (our RNA-seq data). We find that loss of both UTX and JMJD3 leads to a block in NKT development in the thymus.

Like many chromatin modifying enzymes, UTX and JMJD3 do not possess DNA- or histone- binding domains, and are recruited to chromatin by sequence specific transcription factors [23]. UTX associates with T-box transcription factors (T-bet and Eomes in T cells) and promotes gene expression by histone demethylation or through BRG1 recruitment [21]. Initially we hypothesized that failure to express T-bet or T-bet targets may be responsible for the block in NKT development. However we found that T-bet protein expression was similar in WT and UTX/JMJD3 DKO mice. Furthermore, the defect in NKT development observed in Tbet KO mice occurs at the P2 to P3 transition while loss of UTX and JMJD3 causes an earlier arrest. Therefore, although loss of UTX probably inhibits Tbet function in NKT cells as seen in other systems, the developmental block in NKT cells of UTX and UTX/JMJD3 deficient mice likely results from effects on other genes.

The phenotype of the UTX/JMJD3 DKO mouse closely resembles the phenotype of mice in which c-myc has been deleted by CD4-Cre [14]. In these mice, conventional T cell development is largely unaffected while there is a block in NKT development at the P1 to P2 stages. Our RNA-seq showed a modest but consistent reduced level of c-myc in DKO NKT P1 cells. Additionally, the c-myc target gene E2F1, which is expressed by WT cells, was not detected in our KO samples. Similar to Mycko et al. we could not find a cell cycle defect in the NKT cells of UTX/JMJD3 DKO mouse, but it may be that an upstream progenitor which is too rare to detect is rapidly cycling, and these cell divisions do not occur in the UTX/JMJD3 DKO or c-myc KO strains. The signals that direct proliferation of developing NKT cells are not entirely clear, so whether UTX regulates a positive mediator of proliferation, such as a cytokine receptor, or proliferation directly is difficult to know.

Our finding that UTX and EZH2 play antagonistic roles in NKT development suggests a common mechanism, but is not conclusive. An example of this type of regulation occurs in breast cancer cells. In this context, loss of EZH2 alleviated the need for JMJD3 to remove H3K27me3 from an enhancer and activate gene expression of BCL2 [47]. It is also possible that they influence different sets of genes and when both are deleted, these independent effects negate each other. Since the demethylase deficient homolog, UTY, is not sufficient for NKT development, and H3K27me3 deposition appears to be an influential factor, it is likely that EZH2 and UTX/JMJD3 operate on histones located in the same DNA sequences. Determining the identity of these genes will provide a mechanistic link between H3K27 methylation and NKT development.

We hope to determine the effects on H3K27me3 distribution caused by loss of UTX and JMJD3 during NKT development. Unfortunately, conventional ChIP-seq methods that would directly address this question require large numbers of cells. Even sensitive methods that are reported to work on thousands of cells are difficult to perform in this instance due to the extreme rarity of the relevant populations. We hope to perform these experiments in the future.

## Conclusions

The list of factors that distinguish NKT and T cell development is rapidly growing. Because NKT cells are an innate like early responder and shape adaptive immune responses, they are potential therapeutic targets. In this report we describe the critical requirement for UTX and JMJD3 in NKT development. Our results suggest that inhibition of EZH2 may increase NKT cell numbers, and then could help to treat cancer [48]. Alternatively, inhibitors of UTX or JMJD3, such as the recently developed GSK-J4 [49], may block the development of NKT cells, and be used to treat NKT mediated inflammation. In conclusion, NKT development is an interesting model system with clear outcomes to explore the epigenetic control of lineage outcomes.

## Additional files

**Additional file 1: Table S1.** List of antibodies used in WB and FACS analysis.

**Additional file 2: Figure S1.** Deletion of UTX/JMJD3, EZH2, or UTX/EZH2 alters global H3K27me3 levels in CD4 T cells. (A) Western blot of H3 and H3K27me1, me2, and me3 in WT and UTX/JMJD3 DKO samples. Protein levels were observed with film, then scanned and presented without manipulation. All bands are approximately 17 kDa in size, and only a small area around this band is presented. Each blot was done independently by loading equal volumes of a single sample. (B) Western blot of H3 and H3K27me3 in WT, UTX/JMJD3 DKO, UTX/EZH2 DKO, and EZH2 KO CD4 T cells. Signal was collected as in A. Each blot was done independently by loading equal volumes from the same sample. (C) Band intensity for H3K27me3 and total H3 was quantified using Image J software. For UTX/JMJD3 and WT samples we analyzed 3 biological replicates, and for EZH2 and UTX/EZH2 we analyzed 2 biological replicates.

**Additional file 3: Figure S2.** There is an increase in frequency of tetramer^+^ cells in the thymuses of EZH2 KO and EZH2/UTX DKO mice when compared to control mice. (A) Thymocytes from the indicated genotype were isolated and stained for the indicated surface markers. The frequency of tetramer positive cells among many mice was recorded. (B) Quantification of all experiments with numbers for each genotype showing the difference in frequency of tetramer positive thymocytes. The difference between WT and EZH2 KO is significant by two-tailed T test (p < 0.05). There is also a significant difference between WT and UTX/EZH2 DKO (p < 0.05).

**Additional file 4: Table S2.** List of differentially expressed genes between WT and DKO P1 NK T cells.

**Additional file 5: Figure S3.** There is no difference between BRDU or DAPI staining between WT and UTX or UTX/JMJD3 DKO cells. (A) Mice were injected IP with BRDU 12 h before sacrifice. Thymocytes were harvested and stained with surface markers for NKT cells and then the cells were permeablized and stained with BRDU antibodies. No difference was detected between the fraction of tetramer^+^ cells incorporating DAPI in WT and UTX or UTX/JMJD3 DKO mice. Two experiments were done with 5 WT and 3 UTX KO and 2 DKO mice. (B) Negligible DAPI incorporation by Tetramer^+^ cells in the thymus. Cells were stained as in A, and assessed for the incorporation of DAPI. Only a minor fraction of the cells appear to be in S phase, and this is not different between WT and DKO mice. As in A, two experiments were done with 5 WT, 3 UTX KO, and 2 DKO animals.
