# Supplementary material for: Histone demethylases UTX and JMJD3 are required for NKT cell development in mice
Source: Cell Biosci. 2017 May 17;7:25. doi: 10.1186/s13578-017-0152-8 (PMC5436453; doi:10.1186/s13578-017-0152-8)
Supplement: Supplementary file 1 — Additional file 1: Table S1. List of antibodies used in WB and FACS analysis. [file 13578_2017_152_MOESM1_ESM.pdf]

Supplementary Table S1

Reagents	Company	Catalog	Notes
CD4 Monoclonal Antibody (GK1.5)	eBioscience	53-0041-80	Alexa488
Rat Anti-Mouse CD8a Clone 53-6.7 (RUO)	BD Biosciences	562283	PE-CF594
Anti-mouse CD62L	eBioscience	25-0621-82	PE-Cy7
CD44 Monoclonal Antibody (IM7)	eBioscience	56-0441-82	Alexa Fluor 700
Rat Anti-Mouse CD44 Clone IM7 (RUO)	BD Biosciences	553134	PE
CD3 Monoclonal Antibody (17A2)	eBioscience	46-0032-82	PerCP-eFluor 710
CD3e Monoclonal Antibody (145-2C11)	eBioscience	11-0031-85	FITC
CD69 Monoclonal Antibody (H1.2F3)	eBioscience	11-0691-82	FITC
Anti-mouse CD24	eBioscience	25-0242-82	PE-Cy7
NK1.1 Monoclonal Antibody (PK136)	eBioscience	11-5941-82	FITC
NK1.1 Monoclonal Antibody (PK136)	eBioscience	12-5941-82	PE
anti-mouse NK-1.1 Antibody	BioLegend	108733	Brilliant Violet 570™
CD45.1 Monoclonal Antibody (A20)	eBioscience	25-0453-82	PE-Cyanine7
CD45.1 Monoclonal Antibody (A20)	eBioscience	45-0453-80	PerCP-Cyanine5.5
CD45.2 Monoclonal Antibody (104)	eBioscience	47-0454-82	APC-eFluor 780
mCD1d/PBS-57	NIH Tetramer Core Facility	20914	APC-Labeled Tetramer
mCD1d/Unloaded	NIH Tetramer Core Facility	20915	APC-Labeled Tetramer
Anti-H3K27me1	Upstate	07-448	
Anti-H3K27me2	Abcam	ab24684	
Anti-H3K27me3	Upstate	07-449	
