# Supplementary figures and images for: Histone demethylases UTX and JMJD3 are required for NKT cell development in mice
Source: Cell Biosci. 2017 May 17;7:25. doi: 10.1186/s13578-017-0152-8 (PMC5436453; doi:10.1186/s13578-017-0152-8)

Supplemental figure 1

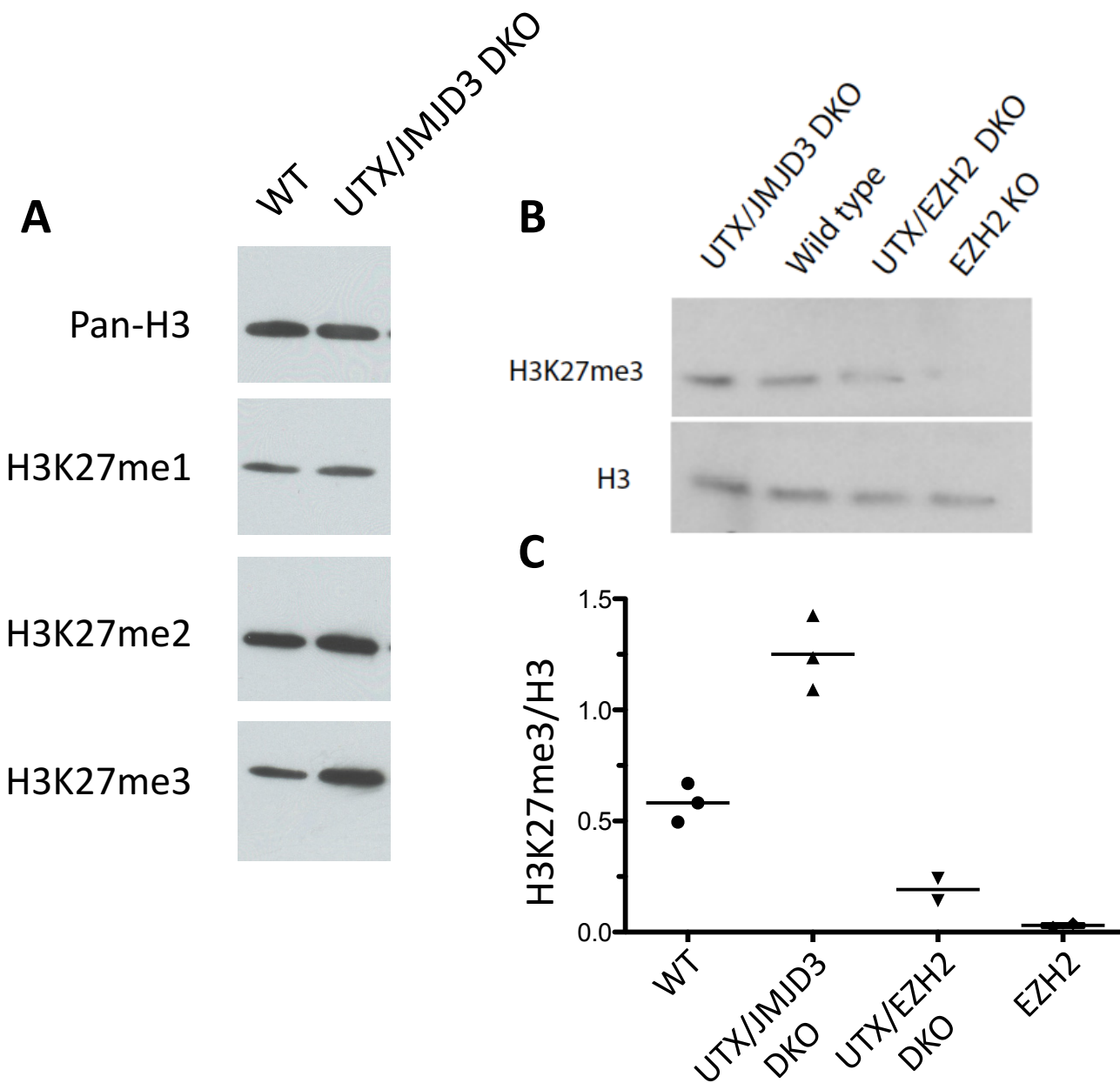

Supplement: Supplementary file 2 — Additional file 2: Figure S1. Deletion of UTX/JMJD3, EZH2, or UTX/EZH2 alters global H3K27me3 levels in CD4 T cells. (A) Western blot of H3 and H3K27me1, me2, and me3 in WT and UTX/JMJD3 DKO samples. Protein levels were observed with film, then scanned and presented without manipulation. All bands are approximately 17 kDa in size, and only a small area around this band is presented. Each blot was done independently by loading equal volumes of a single sample. (B) Western blot of H3 and H3K27me3 in WT, UTX/JMJD3 DKO, UTX/EZH2 DKO, and EZH2 KO CD4 T cells. Signal was collected as in A. Each blot was done independently by loading equal volumes from the same sample. (C) Band intensity for H3K27me3 and total H3 was quantified using Image J software. For UTX/JMJD3 and WT samples we analyzed 3 biological replicates, and for EZH2 and UTX/EZH2 we analyzed 2 biological replicates. [file 13578_2017_152_MOESM2_ESM.pdf]

Supplemental figure 2

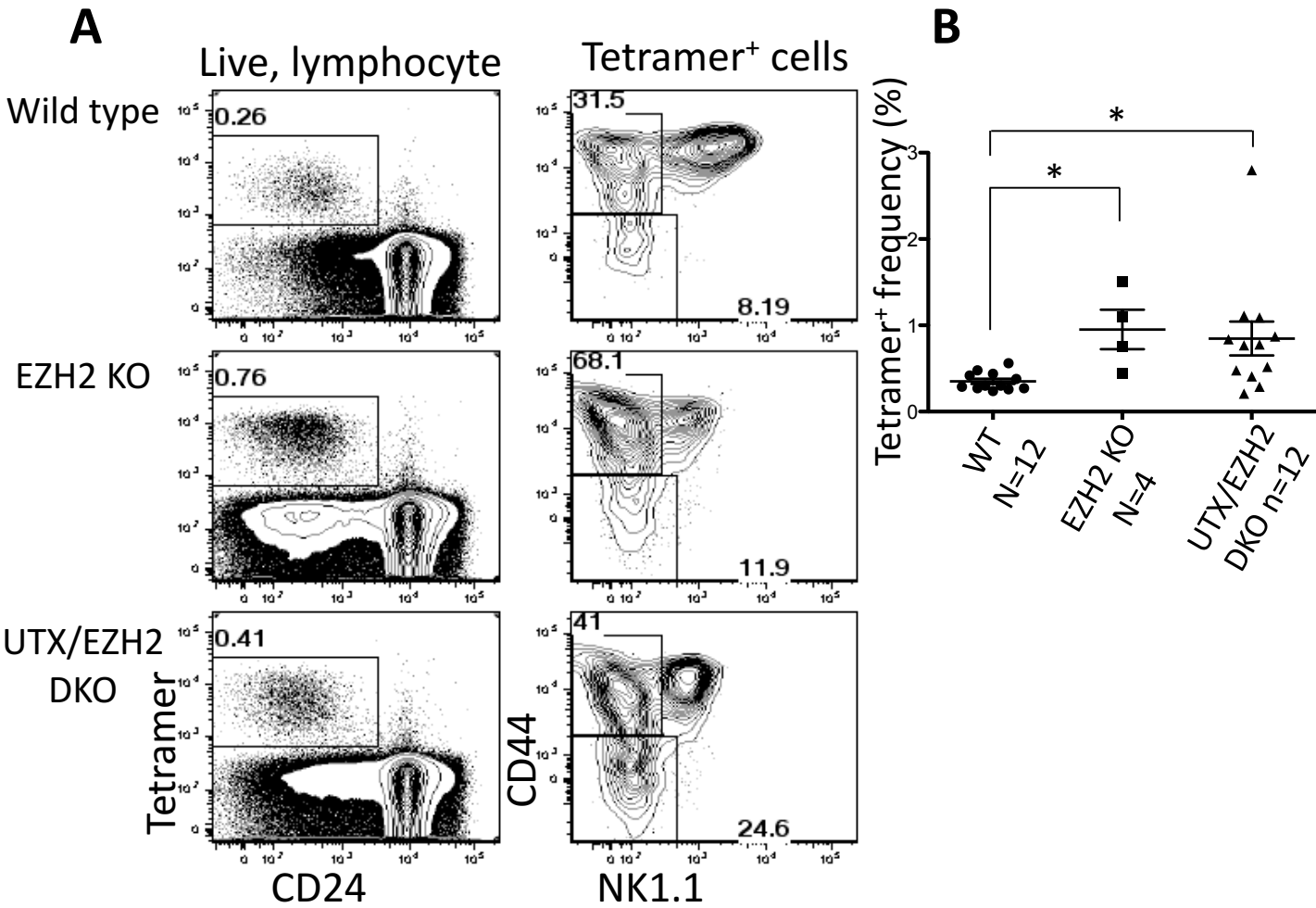

Supplement: Supplementary file 3 — Additional file 3: Figure S2. There is an increase in frequency of tetramer+ cells in the thymuses of EZH2 KO and EZH2/UTX DKO mice when compared to control mice. (A) Thymocytes from the indicated genotype were isolated and stained for the indicated surface markers. The frequency of tetramer positive cells among many mice was recorded. (B) Quantification of all experiments with numbers for each genotype showing the difference in frequency of tetramer positive thymocytes. The difference between WT and EZH2 KO is significant by two-tailed T test (p < 0.05). There is also a significant difference between WT and UTX/EZH2 DKO (p < 0.05). [file 13578_2017_152_MOESM3_ESM.pdf]

Supplemental figure 3

**A**

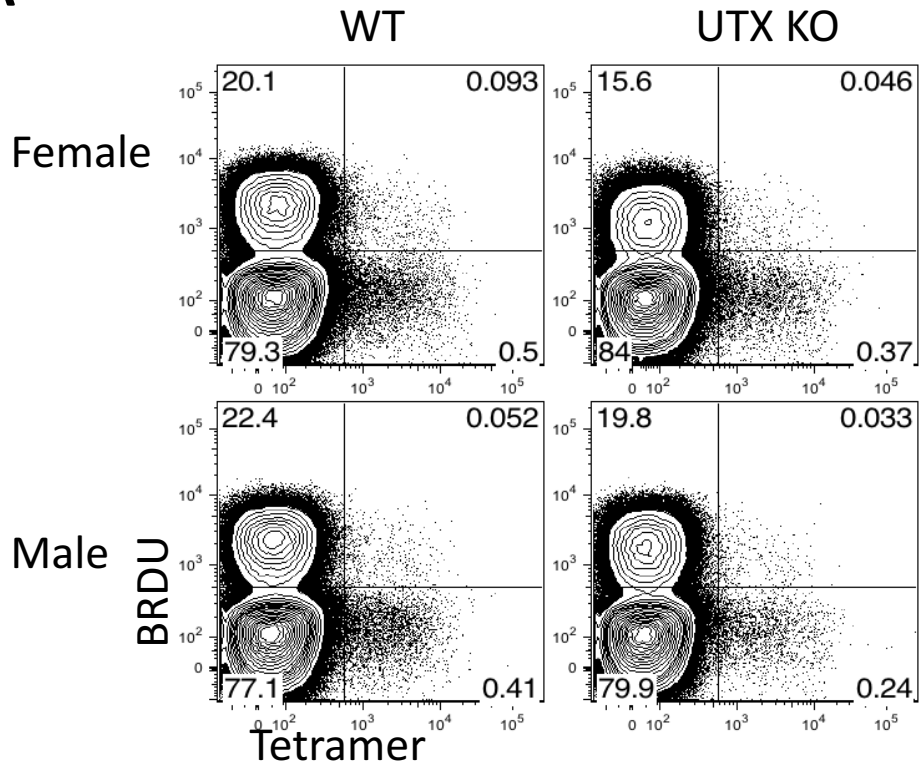

**B**

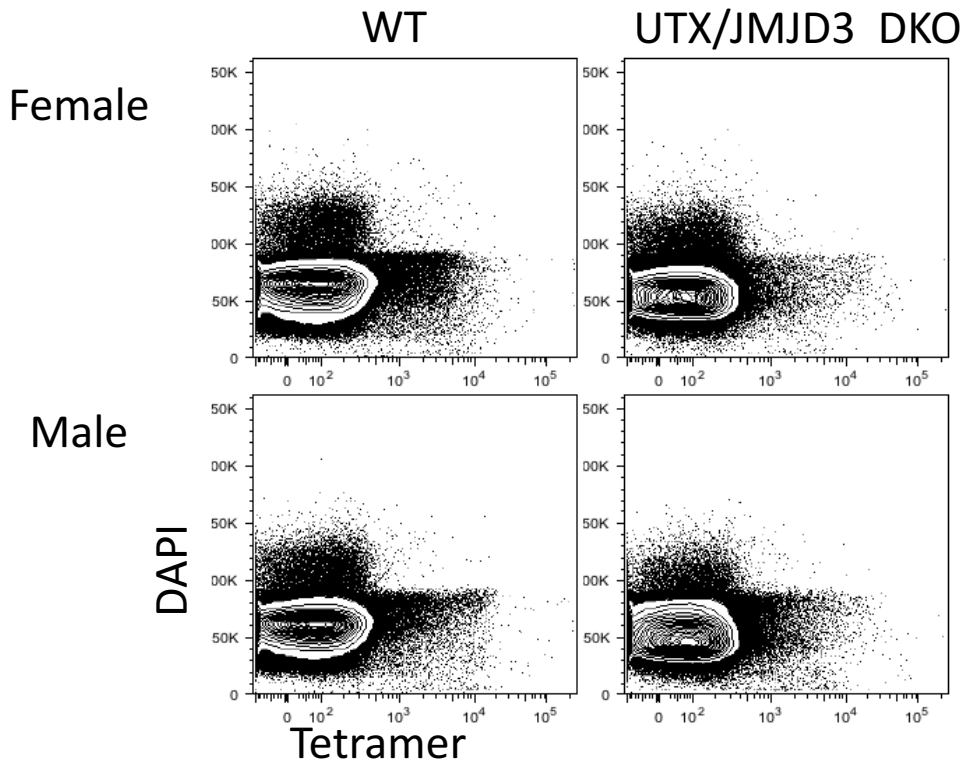

Supplement: Supplementary file 5 — Additional file 5: Figure S3. There is no difference between BRDU or DAPI staining between WT and UTX or UTX/JMJD3 DKO cells. (A) Mice were injected IP with BRDU 12 h before sacrifice. Thymocytes were harvested and stained with surface markers for NKT cells and then the cells were permeablized and stained with BRDU antibodies. No difference was detected between the fraction of tetramer+ cells incorporating DAPI in WT and UTX or UTX/JMJD3 DKO mice. Two experiments were done with 5 WT and 3 UTX KO and 2 DKO mice. (B) Negligible DAPI incorporation by Tetramer+ cells in the thymus. Cells were stained as in A, and assessed for the incorporation of DAPI. Only a minor fraction of the cells appear to be in S phase, and this is not different between WT and DKO mice. As in A, two experiments were done with 5 WT, 3 UTX KO, and 2 DKO animals. [file 13578_2017_152_MOESM5_ESM.pdf]
